# Ultrasound irradiation activates purine metabolism and mitochondrial respiration via the MAPK signaling pathway in myotubes

**DOI:** 10.1016/j.bbrep.2025.101984

**Published:** 2025-03-26

**Authors:** Xiaoqi Ma, Noriaki Maeshige, Atomu Yamaguchi, Yunfei Fu, Jihao Xing, Qingcheng Guo, Hao Lin, Fuwen Lu, Hiroyo Kondo, Hidemi Fujino

**Affiliations:** a Department of Rehabilitation Science, Kobe University Graduate School of Health Sciences, Kobe, Japan; b Shanghai Yangzhi Rehabilitation Hospital (Shanghai Sunshine Rehabilitation Center), Tongji University School of Medicine, Shanghai, China; c Faculty of Health and Nutrition, Shubun University, Ichinomiya, Japan

**Keywords:** Myotubes, Pulsed ultrasound, MAPK signaling pathway, Purine metabolism, Mitochondrial respiration

## Abstract

**Background:**

Pulsed ultrasound (US) is widely used both as a diagnostic imaging tool and a therapeutic approach. However, many of the mechanisms underlying the therapeutic effects of non-thermal US remain unclear, especially in skeletal muscles, which play a crucial role in the body's metabolism. The aim of this study was to investigate the effects of US on myotubes.

**Methods:**

In this study, C2C12 myoblasts were utilized. After differentiating into myotubes, the cells were exposed to US irradiation at an intensity of 3.0 W/cm^2^, with a 20 % duty cycle, an acoustic frequency of 1 MHz, and a pulse repetition frequency of 100 Hz for 5 min. The cells were then collected and analyzed for genomic and metabolomic alterations, as well as mitochondrial function.

**Results:**

Cell viability remained unaffected after US irradiation. The mitogen-activated protein kinase (MAPK) signaling pathway was the most activated, while the expression of various RNAs was significantly altered. Purine metabolism was highly activated, with an increase in the abundance of metabolites associated with this pathway. Furthermore, mitochondrial respiration in the myotubes increased following US irradiation.

**Conclusion:**

This study investigated the impact of US irradiation on myotubes using genomic analysis, metabolomic analysis, and mitochondrial function. US irradiation activated the MAPK signaling pathway, which in turn enhanced purine metabolism and improved mitochondrial respiration.

## Highlights

• Ultrasound irradiation activated purine metabolism without increasing the expression of uric acid.
• Ultrasound irradiation increased mitochondrial respiration in myotubes.
• Ultrasound irradiation activated the MAPK signaling pathway in myotubes.

## 1 Introduction

Pulsed ultrasound (US) is extensively utilized not only for diagnostic imaging but also as a non-invasive, painless, and safe therapeutic approach, rendering it suitable for a diverse range of patients [1]. With a long history, the non-thermal impacts of US have attracted increasing attention [2]. Its applications in alleviating pain, promoting tissue healing, and improving tissue characteristics have grown in popularity [[1], [2], [3]]. Therapeutic US is commonly used to treat muscle injuries [4], has anti-inflammatory effects [5], and prevents muscle atrophy [6]. Despite the plethora of potential therapeutic applications, the mechanisms underlying the non-thermal effects of US remain largely unexplored and are still under investigation [2].

Skeletal muscle is the largest organ in the human body, accounting for about 40 % of total body mass [7,8]. It contributes approximately 30 % to the resting metabolic rate and is implicated in up to 75 % of total body metabolism [9]. It plays a crucial role in glucose uptake and is pivotal in both exercise and metabolic disease [10]. Skeletal muscle is also vital for regulating its own metabolism, particularly in response to exercise and pathological conditions [[11], [12], [13]]. The mitogen-activated protein kinase (MAPK) signaling pathway plays an essential role in cellular metabolism [14]. Mitochondrial function contributes significantly to muscle metabolism [15]. Mitochondria are essential organelles in cellular metabolism that play a key role in regulating the metabolic state of skeletal muscles [15,16]. Therefore, studying the intricate roles of the MAPK signaling pathway and mitochondrial function in skeletal muscle metabolism is vital for advancing our knowledge of metabolic regulation and potential therapeutic interventions.

As US irradiation regulates the MAPK pathway [17], which influences skeletal muscle metabolic alterations, we hypothesized that US regulates the skeletal muscle metabolic state through the MAPK signaling pathway. Given the unclear effects of US irradiation on metabolites and their mechanisms in skeletal muscle, this study aimed to determine the effects of US on myotubes using genomic analysis, metabolomic analysis, and mitochondrial function.

## 2 Materials and methods

### 2.1 Cell culture

C2C12 myoblasts, mouse skeletal muscle cells, were obtained from the American Type Culture Collection (ATCC). These cells were cultured in 35-mm dishes containing Dulbecco's modified Eagle medium (DMEM) supplemented with 10 % fetal bovine serum (FBS) at 37 °C under 5 % CO_2_. The growth medium was replaced with a differentiation medium consisting of DMEM supplemented with 2 % horse serum (HS) at approximately 90 % confluence. The cells were cultured for 6 or 7 days to allow differentiation. After differentiation, US irradiation was applied to the myotubes.

### 2.2 US irradiation

Following cell differentiation, US irradiation was performed for 5 min using a medical US device (SZ-100 M; MINATO Medical Science 129 Co., LTD, Japan). The device probe was placed beneath the bottom of the culture dish, and a coupling gel was used between the culture dish and probe. A piece of sterilized silicone was suspended 2 mm above the cell monolayer in culture media [18]. US parameters were as follows: intensity, 3.0 W/cm^2^; duty cycle, 20 %; acoustic frequency, 1 MHz; and repetition frequency, 100 Hz. The beam nonuniformity ratio, which represents the ratio of the maximum intensity to the average intensity of the US, was 2.4, confirming that the probe was safe. The temperature of the culture medium was below 37 °C during the US irradiation.

### 2.3 Cell viability analysis

In this study, myotube viability was evaluated using the MTT assay after US irradiation. Cells were incubated with 5 mg/mL MTT solution (10 × ) and 3-(4,5-dimethyl-2-thiazolyl)-2,5-diphenyl-2 H-tetrazolium bromide (Wako Junyaku Co., Ltd., Japan) diluted in the culture medium. Following incubation for 3 h, dimethyl sulfoxide (DMSO) was added. The absorbance was measured at 595 nm using an MTP-300 spectrophotometer (Kono Electric Co., Ltd., Japan). Cell viability was expressed as a percentage relative to the CON group.

Myotube viability was also evaluated using Zombie Red™ staining. The cells were rinsed twice with PBS and incubated with the Zombie Red™ Fixable Viability Kit (1:1000; BioLegend, CA, USA) for 15 min. Zombie Red™ is an amine-responsive fluorescent dye that is impermeable to living cells but permeable to those with damaged plasma membranes. Following incubation, the cells were rinsed twice with PBS and incubated with 4 % formaldehyde at room temperature for 30 min. The cell nuclei were counterstained with DAPI (1:1, 000; Dojindo, Kumamoto, Japan) for 5 min. The stained cells were imaged using a fluorescence microscope (BZ-X800; Keyence, Japan).

### 2.4 Reactive oxygen species (ROS) analysis and DNA damage analysis

ROS production was analyzed using CellRox Green Reagent (Invitrogen). The reagent was added to each dish at a concentration of 10 μmol/L, and the cells were incubated for 30 min at 37 °C in a 5 % CO_2_ incubator. The dishes were washed with PBS and incubated with 3.7 % formaldehyde for 15 min at room temperature. DAPI (1:1000; Dojindo) was added, and the cells were incubated for 5 min at 37 °C after washing with PBS. Stained cells were observed under a fluorescence microscope (BZ-X800; Keyence, Japan). The fluorescence intensity of each dish was determined using Image J, and the results were analyzed as a ratio to those of the control group.

TUNEL staining was performed using an in situ Apoptosis Detection kit (Takara In Situ Apoptosis Detection Kit; Takara Bio Inc., Shiga, Japan) to analyze DNA damage in the cells. The cells were then rinsed twice with PBS and incubated with 4 % formaldehyde at room temperature for 30 min. After rinsing the cells twice with PBS, 0.3 % H_2_O_2_ in methanol was added at room temperature. After 30 min, cells were washed twice with PBS, and permeabilization buffer was added for 5 min at 4 °C. The TdT enzyme and labeling safe buffer were then added and incubated at 37 °C for 60 min. The cell nuclei were counterstained with DAPI (1:1, 000; Dojindo, Kumamoto, Japan) for 5 min. The stained cells were imaged using a fluorescence microscope (BZ-X800; Keyence, Japan).

### 2.5 Seahorse assay

To evaluate the bioenergetic condition of the myotubes after US irradiation, a Seahorse XFp Analyzer (Agilent Technologies, USA) was utilized for mitochondrial stress tests to measure the oxygen consumption rate (OCR). C2C12 myoblasts were cultured in XFp cell culture miniplates to differentiate into myotubes. After differentiation, the myotubes were subjected to US irradiation for 5 min. The Seahorse assay was then performed after a 24-h period.

For OCR detection, the medium was replaced with Seahorse XF Assay medium supplemented with 10 mM glucose, 1 mM pyruvate, and 2 mM glutamine 24 h after US irradiation. The miniplate was then incubated at 37 °C in a non-CO_2_ incubator for 30 min. OCRs were measured under basal conditions and after sequential application of 1 μM oligomycin (to assess ATP-related oxygen consumption), 1.5 μM carbonyl cyanide 4-(trifluoromethoxy) phenylhydrazone (FCCP) (to determine the maximum respiratory capacity), and 2 μM rotenone/antimycin A (R/A) mixture (to evaluate the non-mitochondrial oxygen consumption). After OCR was detected, the cells were rinsed once with PBS and lysed in lysis buffer. The lysate was centrifuged to obtain the protein supernatant. The BCA assay was employed to measure the protein concentration. OCR values were normalized to protein concentration and expressed as pmol/min/μg protein.

### 2.6 RNA sequencing of myotubes

Total RNA was extracted from the myotubes using TRIzol reagent (Takara Biotechnology, Japan). Raw RNA sequence data were obtained utilizing an Illumina NovaSeq 6000 machine. The fold change (mean expression of each RNA in the US group divided by the mean expression in the control group) and P value for each RNA were calculated after obtaining the raw data. These P values were then used to determine the false discovery rate (FDR) for each RNA, which was then applied as a filter to identify essential RNAs with an FDR <0.05. RaNA-Seq and the R 3.5.3 program were utilized for pathway enrichment analysis. After performing pathway analysis using the Kyoto Encyclopedia of Genes and Genomes database, the 10 most enriched pathways associated with signal transduction were identified and used to explore related pathways. The RNA-seq datasets produced in this study have been uploaded to the NCBI Sequence Read Archive. The accession number for the dataset is PRJNA1116252. Detailed sequencing data and relevant metadata can be accessed at https://www.ncbi.nlm.nih.gov/search/all/?term=PRJNA1116252.

### 2.7 Metabolite analysis

At 24 h after US irradiation, myotubes were rinsed twice with PBS and then lysed in 80 % methanol containing 50 μM (+)-10-camphorsulfonic acid, 400 μM l-methionine sulfone, and 400 μM piperazine-1,4-bis (2-ethanesulfonic acid) as internal standards. The cells were incubated at −80 °C for 15 min followed by scraping and centrifugation at 14,000 g for 5 min at 4 °C. The supernatants were collected and filtered through a Millipore 5 kDa cut-off membrane to remove solubilized proteins. The dried metabolites were dissolved in Milli-Q water after evaporating the aqueous layer extracts under vacuum using a FreeZone 2.5 Plus freeze-dry system (Labconco, Kansas City, MO). Intracellular metabolite concentrations were analyzed using LC-MS to detect metabolites from a library of 300 targeted compounds, including those in the TCA cycle, 3-phosphoglycerol shuttle, and glycolysis [19]. MetaboAnalyst 5.0, an online software, was used for pathway analysis (https://www.metaboanalyst.ca) [20].

### 2.8 Western blotting

The culture medium was removed, and C2C12 myotubes were washed with PBS, scraped, and homogenized in lysis buffer. Homogenates were centrifuged at 15,000 rpm for 10 min at 4 °C, and the supernatants were collected. The supernatant protein concentrations were determined using a BCA protein assay. Samples were standardized, and equal amounts of protein were separated through 7.5 % or 12.5 % SDS-polyacrylamide gel electrophoresis and transferred to polyvinylidene difluoride membranes. The membranes were blocked with 3 % BSA in TBS with 0.1 % Tween 20 (TBST) or 5 % skim milk in PBS with 0.1 % Tween 20 (PBST). Membranes were incubated with p38 MAPK (1:1000 in TBST, #8690, Cell Signaling), phospho-p38 MAPK (1:1000 in TBST, #4511, Cell Signaling), and GAPDH (1:1000 in PBST, #97166, Cell Signaling) at 4 °C overnight. The membranes were then incubated with horseradish peroxidase-conjugated anti-rabbit or anti-mouse IgG (1:10,000 in TBST; GE Healthcare, Waukesha, WI, USA) for 1 h at room temperature. Membrane proteins were detected using a chemiluminescent reagent (Ez West Lumi; ATTO, Tokyo, Japan) and quantified with an image reader (LAS-1000; Fujifilm, Tokyo, Japan).

### 2.9 Statistical analysis

All values are expressed as means ± standard error of the mean (SEM). Differences were considered statistically significant when p < .05, as determined by a two-tailed Student's t-test.

## 3 Results

### 3.1 US irradiation does not affect cell viability

**Fig. 1:**
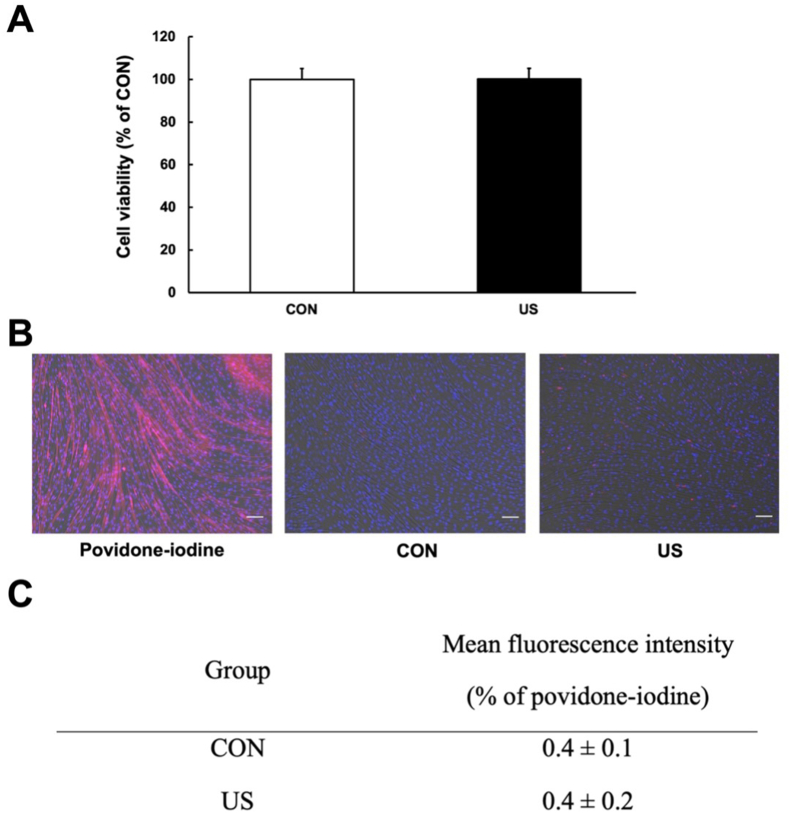
Cell viability. A: MTT assay was performed after ultrasound (US) irradiation to measure cell viability, expressed as a percentage relative to the control. All values are represented as mean ± SEM (n = 3). B: Zombie Red™ immunofluorescence staining (red) in myotubes after US irradiation. After fixation, myotubes were counter-stained with DAPI (blue). Scale bar = 100 μm. C: Cell viability analysis using Zombie Red™ immunofluorescence staining. Mean fluorescence intensity of Zombie Red™ in myotubes. Values are presented as percentage of povidone-iodine and expressed as means ± SEM.

As depicted in Fig. 1A, compared to that in the control group, there was no decrease in cell viability in the US group, suggesting that US irradiation did not reduce cell viability. Additionally, as depicted in Fig. 1B and C, Zombie Red™ immunofluorescence staining was performed to evaluate the cytotoxic effects of US irradiation on myotubes. Treatment with 1 % povidone-iodine significantly reduced cell viability, while US irradiation did not affect cell viability. In addition, we verified whether US irradiation increased ROS expression in myotubes through immunofluorescence staining for ROS and whether it caused DNA damage in myotubes using TUNEL staining. The results showed that US irradiation did not increase ROS expression in myotubes (Supplementary File, Fig. 1) or cause DNA damage (Supplementary File, Fig. 3).

### 3.2 US irradiation most effectively activated the MAPK signaling pathway

**Fig. 2:**
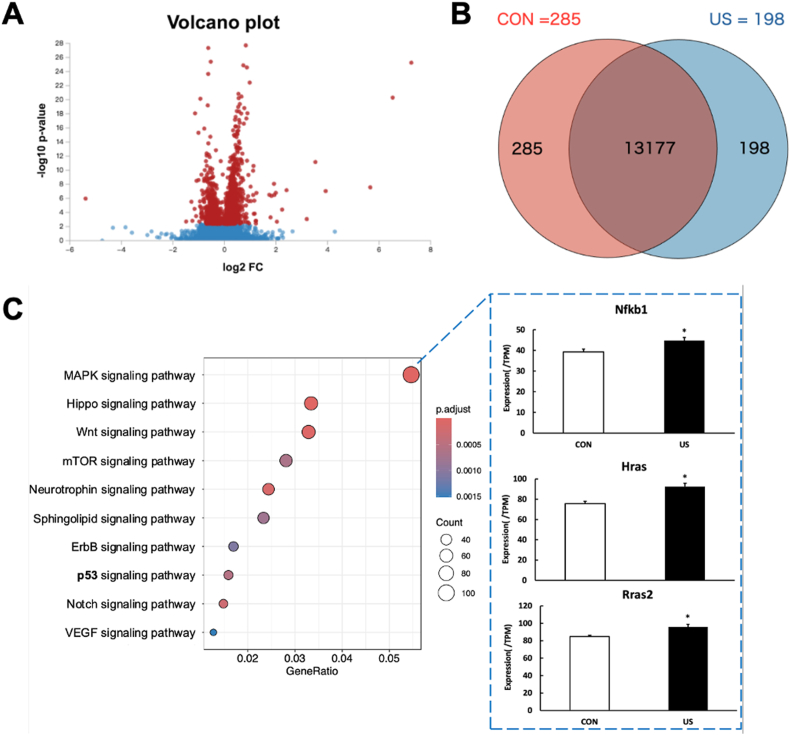
RNA sequencing analysis of myotubes after ultrasound (US) irradiation. A: Volcano plot of differentially expressed RNAs in control group vs. US group. Red dots represent RNAs with statistically significant difference, and blue dots indicate RNAs with no statistically significant difference between the US and control groups. B: A Venn diagram showing unique and overlapping differentially expressed genes (DEGs) between US and control groups. C: Kyoto Encyclopedia of Genes and Genomes (KEGG) analysis was performed on differentially expressed RNAs, with the 10 most enriched pathways linked to signaling transduction presented (n = 3).

RNA sequencing was performed to determine the mechanisms activated by US irradiation in myotubes after US irradiation. A total of 13,660 RNAs were identified using quantitative proteomic analysis. Differentially expressed RNAs were visualized using a volcano plot (Fig. 2A). Two hundred and eighty-five mRNAs were specifically expressed in the control group, while 198 mRNAs were specifically expressed in the US group (Fig. 2B). Enrichment analysis revealed the ten most activated signaling pathways (Fig. 2C). Among these pathways, the MAPK signaling pathway was the most activated. Furthermore, the expression levels of three factors (Nfkb1, Hras, and Rras2) were significantly upregulated in this pathway.

**Fig. 3:**
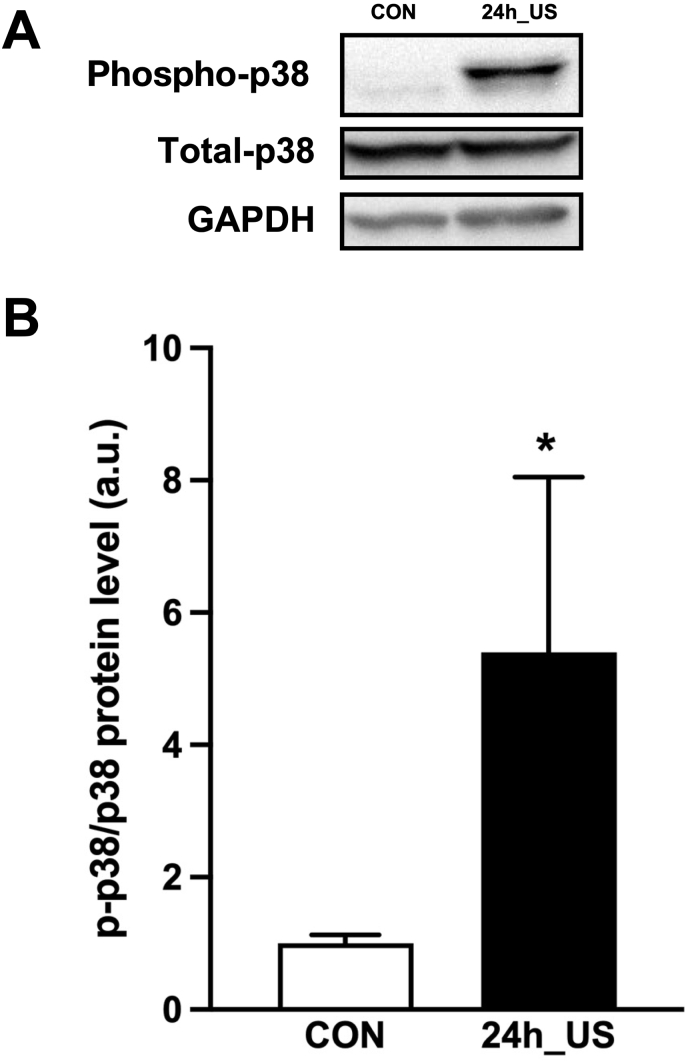
Effect of US irradiation on p38 MAPK in myotubes. A: Representative western blots of Phospho-p38, Total-p38 and GAPDH. B: The phosphorylation and total protein levels of p38 MAPK were measured in this study.

### 3.3 US irradiation induces phosphorylation of p38 MAPK in myotubes

To assess MAPK activation at the protein level, we specifically measured the activation of phospho-p38, a key kinase in the MAPK pathway, 24 h after US irradiation in myotubes. Western blotting analysis revealed a significant increase in the phosphorylation level of p38 MAPK (phospho-p38), while the protein levels of GAPDH and total-p38 remained unchanged (Fig. 3A and B, Supplementary File, Fig. 4).

**Fig. 4:**
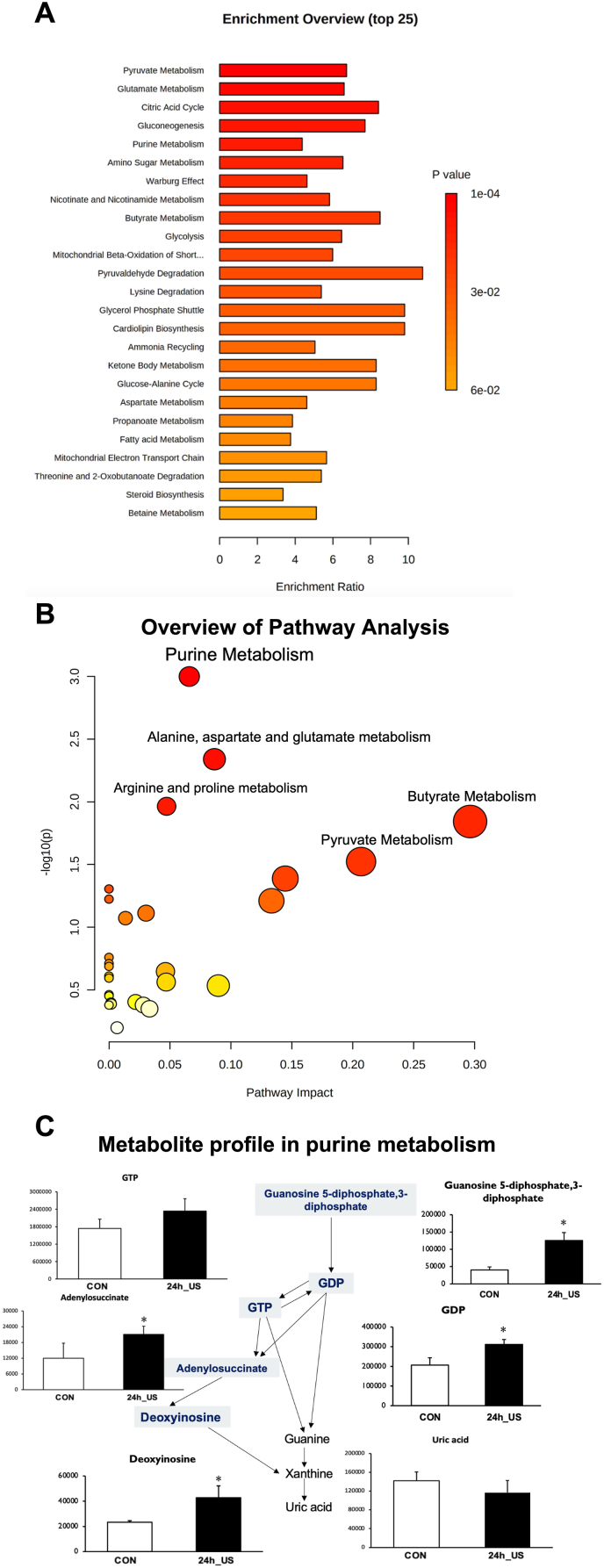
Metabolite analysis of myotubes after ultrasound (US) irradiation. A: Overview of enrichment analysis of myotubes after US irradiation. B: Overview of pathway analysis of myotubes after US irradiation. C: The abundance of metabolites in purine metabolism. All values are represented as mean ± SEM.∗p < .05 vs. control (n = 3).

### 3.4 US irradiation activates purine metabolism in myotubes

To study the metabolite profile of the myotubes after US irradiation, the abundance of metabolites in the myotubes was quantified. To investigate the potential metabolic pathways affected in myotubes after US irradiation, enrichment and pathway analyses were conducted using MetaboAnalyst 5.0 online software (Fig. 4). As depicted in Fig. 4A and B, US irradiation significantly activated several metabolic pathways in myotubes, including pyruvate metabolism, glutamate metabolism, the citric acid cycle, glycogenesis, and purine metabolism, with purine metabolism showing the most significantly activation. Within purine metabolism, the levels of guanosine 5-diphosphate, 3-diphosphate, GDP, deoxyinosine, and adenylosuccinate were significantly elevated, while the expression level of uric acid remained unchanged (Fig. 4C).

### 3.5 US irradiation enhances mitochondrial respiration in myotubes

**Fig. 5:**
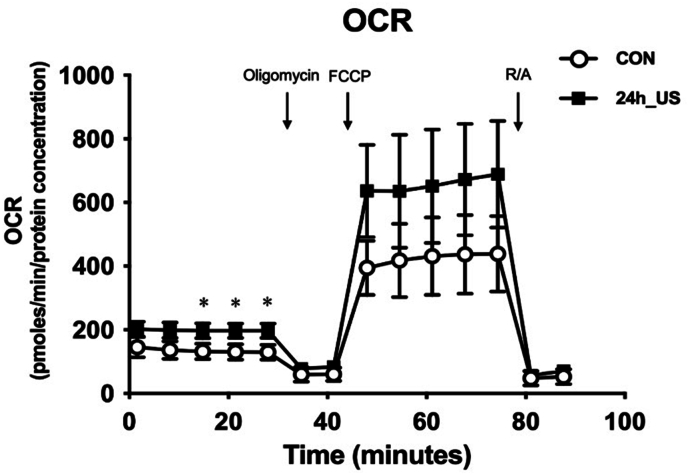
Seahorse analysis of myotubes after ultrasound (US) irradiation. All values are presented as mean ± SEM.∗p < .05 vs. control (n = 3).

To determine the effects of US irradiation on mitochondrial respiration in myotubes, we performed a seahorse assay. Fig. 5 illustrates the changes in the OCR of myotubes upon successive addition of oligomycin, FCCP, rotenone/succinate, and antimycin A during the experiment. Compared to the control group, US irradiation significantly increased the mitochondrial basal respiration rate. In addition, a tendency towards increased maximal mitochondrial respiration was observed.

## 4 Discussion

In the present study, we investigated the effects of US irradiation on myotubes. The results of cell viability analysis indicated that US irradiation did not induce cell damage. Furthermore, DNA damage was detected in the myotubes after US irradiation. However, the results indicated that US irradiation did not increase DNA damage, confirming the safety of US, which is consistent with previous research [21].

In the present study, US irradiation activated various types of RNAs, with the MAPK signaling pathway showing the greatest activation. In addition, western blotting revealed that MAPK was activated at the protein level 24 h post-US. US irradiation reportedly affects the MAPK signaling pathway [22]. The findings of this study also indicated that the effects and changes induced by US irradiation in myotubes are mainly mediated through the activation of this pathway. Furthermore, the expression of Nfkb1, Hras, and Rras2 in the MAPK pathway was significantly upregulated. Hras and Rras2 are members of the RAS small GTPase family [23]. RAS interacts with various downstream factors such as PI3K to affect purine metabolism [24]. Activation of the MAPK signaling pathway can upregulate purine biosynthesis by increasing the expression of purine metabolic enzymes [25,26]. Thus, we believe that US stimulates purine metabolism in myotubes through MAPK pathway activation. Purine metabolism encompasses pathways for both the synthesis and degradation of purines, which are key components of cellular energy systems, signaling molecules, and nucleic acids. Disruptions in purine metabolism can affect purine catabolism, nucleotide synthesis, and salvage pathways [27]. Activation of purine metabolism helps meet increased energy demands, ensures a sufficient supply for cells, and enhances intracellular signaling processes, all of which are essential for cell growth, proliferation, and overall metabolic efficiency [27,28]. Uric acid, the end product of purine catabolism, can accumulate and contribute to disorders like gout [27]. In this study, the expression levels of guanosine 5-diphosphate, 3-diphosphate, GDP, deoxyinosine, and adenylosuccinate were significantly increased in myotubes irradiated with US, which is beneficial for cell growth and cellular energy metabolism [29,30]. Simultaneously, uric acid expression did not increase, indicating that US irradiation had no pathological effect on the myotubes despite the activation of purine metabolism.

In contrast, activation of the Ras cascade enhances the mitochondrial content of respiratory enzymes, thereby enhancing respiratory competence [31]. NFkB activation can promote mitochondrial respiration [32]. Our study demonstrated that US irradiation significantly upregulated the expression levels of Nfkb1, Hras, and Rras2 and increased the mitochondrial basal respiration rate in myotubes without increasing DNA damage and ROS expression. This suggests that US irradiation can safely enhance mitochondrial respiration by activating the MAPK signaling pathway in myotubes. Skeletal muscles with higher mitochondrial respiratory capacity can meet increased energy demands during exercise, allowing muscles to sustain higher workloads for longer periods before fatigue sets in Ref. [33]. Furthermore, strong mitochondrial function helps cells manage stresses such as oxidative damage, calcium overload, and metabolic disturbances, offering protection against mitochondrial dysfunction linked to diseases and aging [34,35]. Therefore, we propose that US irradiation has the potential to enhance muscle function.

In addition to the MAPK signaling pathway, the analysis suggests that the Hippo and Wnt signaling pathways were also be activated, as indicated by the changes in mRNA expression levels observed in this study. YAP and Beta Catenin are the predominant factors in these pathways [36], and the expression of YAP was significantly increased in the Hippo signaling pathway in this study (Supplementary File, Fig. 2). While mRNA expression changes can provide insights into the potential activation of these pathways, it is important to note that mRNA levels alone do not directly confirm the activation of the associated protein factors. Previous studies have shown that while YAP and Beta Catenin are transcriptional factors involved in cell growth and can promote neoplastic transformation if deregulated, previous studies have indicated that YAP's activity may lead to increased nuclear localization and promote cell growth without necessarily causing malignant transformation, unless combined with additional oncogenic signals [36,37]. In summary, we believe that the conditions provided by the US irradiation used in this study were insufficient to induce neoplastic transformation and further experiments, including protein-level analysis, will be necessary to more definitively assess the activation of these pathways.

While the applications of US have traditionally centered on imaging and physiotherapeutic purposes, in this study, our exploration of its influence on muscle cells revealed a previously unknown dimension of cellular responses. We found that US irradiation promoted purine metabolism and mitochondrial respiration by activating the MAPK signaling pathway, benefiting muscle function by supporting cellular energy and protecting against stress. These findings regarding the effects of US on cellular functions significantly contribute to the expanding body of knowledge in this area, offering valuable insights with far-reaching implications for both clinical practice and research applications.

## 5 Conclusion

This study illustrated the effects of US on cultured myotubes in terms of genomic analysis, metabolomic analysis, and mitochondrial function. US irradiation can trigger the MAPK signaling pathway, thereby activating purine metabolism and improving mitochondrial respiration. Thus, US irradiation is an effective method for boosting skeletal muscle function.

## CRediT authorship contribution statement

**Xiaoqi Ma:** Writing – review & editing, Writing – original draft, Visualization, Methodology, Funding acquisition, Formal analysis, Data curation, Conceptualization. **Noriaki Maeshige:** Writing – review & editing, Supervision, Resources, Funding acquisition, Formal analysis, Data curation, Conceptualization. **Atomu Yamaguchi:** Writing – review & editing, Visualization, Methodology, Formal analysis, Data curation, Conceptualization. **Yunfei Fu:** Writing – review & editing, Visualization, Data curation. **Jihao Xing:** Writing – review & editing, Visualization, Data curation. **Qingcheng Guo:** Writing – review & editing, Data curation, Investigation. **Hao Lin:** Writing – review & editing, Visualization, Data curation. **Fuwen Lu:** Writing – review & editing, Data curation. **Hiroyo Kondo:** Writing – review & editing, Resources. **Hidemi Fujino:** Writing – review & editing, Supervision, Resources, Formal analysis, Data curation.

## Data availability statement

The datasets used and analyzed in this study are available upon request from the corresponding author.

## Ethical statement

This study was approved by the Institutional Animal Care and Use Committee and performed according to the Kobe University Animal Experimentation Regulations.

## Declaration of competing interest

The authors declare that they have no known competing financial interests or personal relationships that could have appeared to influence the work reported in this paper.
