# Supplementary material for: Ultrasound irradiation activates purine metabolism and mitochondrial respiration via the MAPK signaling pathway in myotubes
Source: Biochem Biophys Rep. 2025 Mar 26;42:101984. doi: 10.1016/j.bbrep.2025.101984 (PMC11986604; doi:10.1016/j.bbrep.2025.101984)
Supplement: Multimedia component 1 [file mmc1.docx]

**Supplementary File**

**Fig. 1**

**
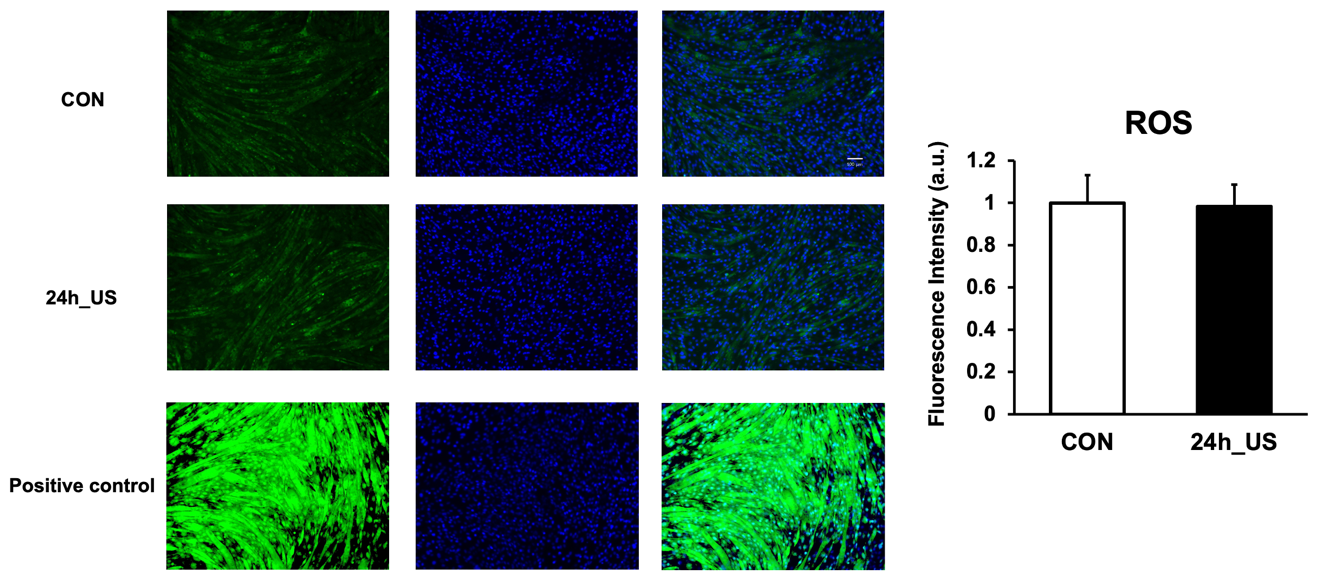
**

Reactive oxygen species (ROS) generation. Myotubes at 24h post-US were stained (lower) compared to the CON group (higher). The fluorescence intensities of each group were calculated by Image J. The intensity of the US group was not significantly higher than that of the CON group. Blue: DAPI; Green: ROS. The statistical differences between these groups were tested by Tukey–Kramer test (n = 3). Scale bar =100 μm. Positive control: Myotubes treated with 2mM H_2_O_2_ for 30 minutes prior to staining, inducing ROS production and serving as a reference for maximum detectable ROS levels in this assay.

**Fig. 2**

**
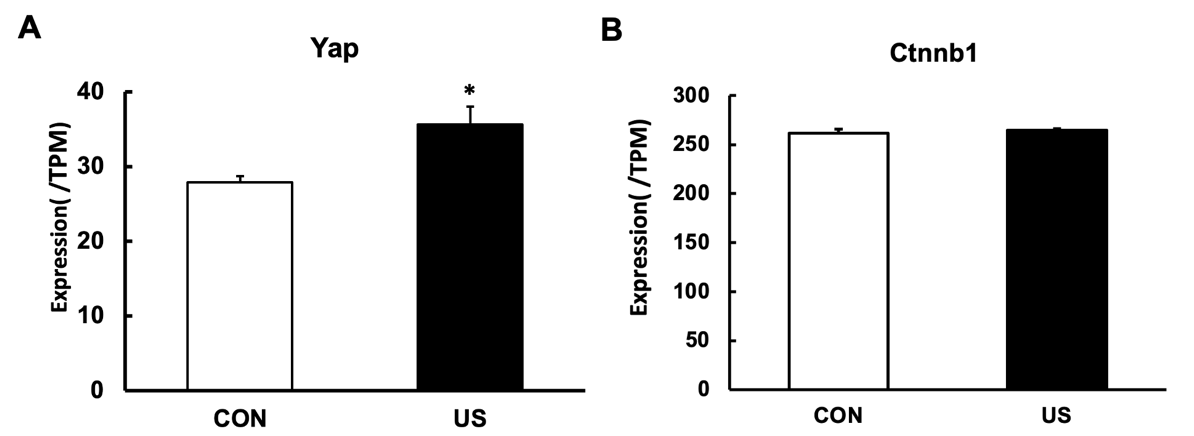
**

The expression of Yap and Ctnnb1 by RNA-seq. A: The expression of Yap. B: The expression of Ctnnb1.

**Fig. 3**


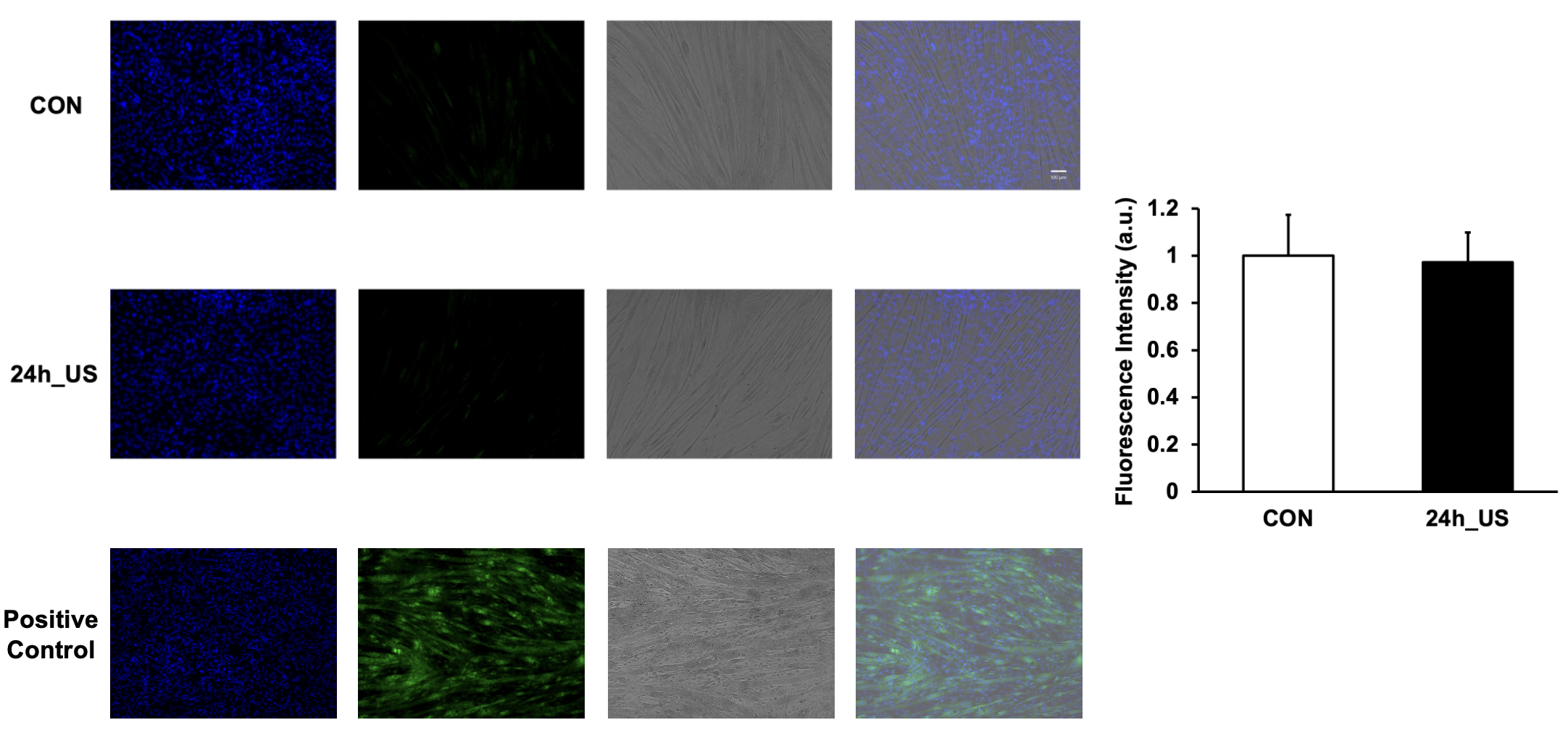


DNA damage by TUNEL staining. Myotubes were stained with DAPI and TUNEL. Myotubes at 24h post-US were stained (lower) compared to the CON group (higher). The fluorescence intensities of each group were calculated by Image J. The intensity of the US group was not significantly higher than that of the CON group, which means the DNA damage did not increase by the US irradiation. Blue: DAPI; Green: TUNEL. The statistical differences between these groups were tested by Tukey–Kramer test (n = 3). Scale bar =100 μm. Positive control: Myotubes treated with 2 mM H_2_O_2_ for 30 minutes prior to staining, inducing DNA fragmentation and apoptosis, serving as a reference for maximum detectable DNA strand breaks in this assay.

**Fig. 4**

**
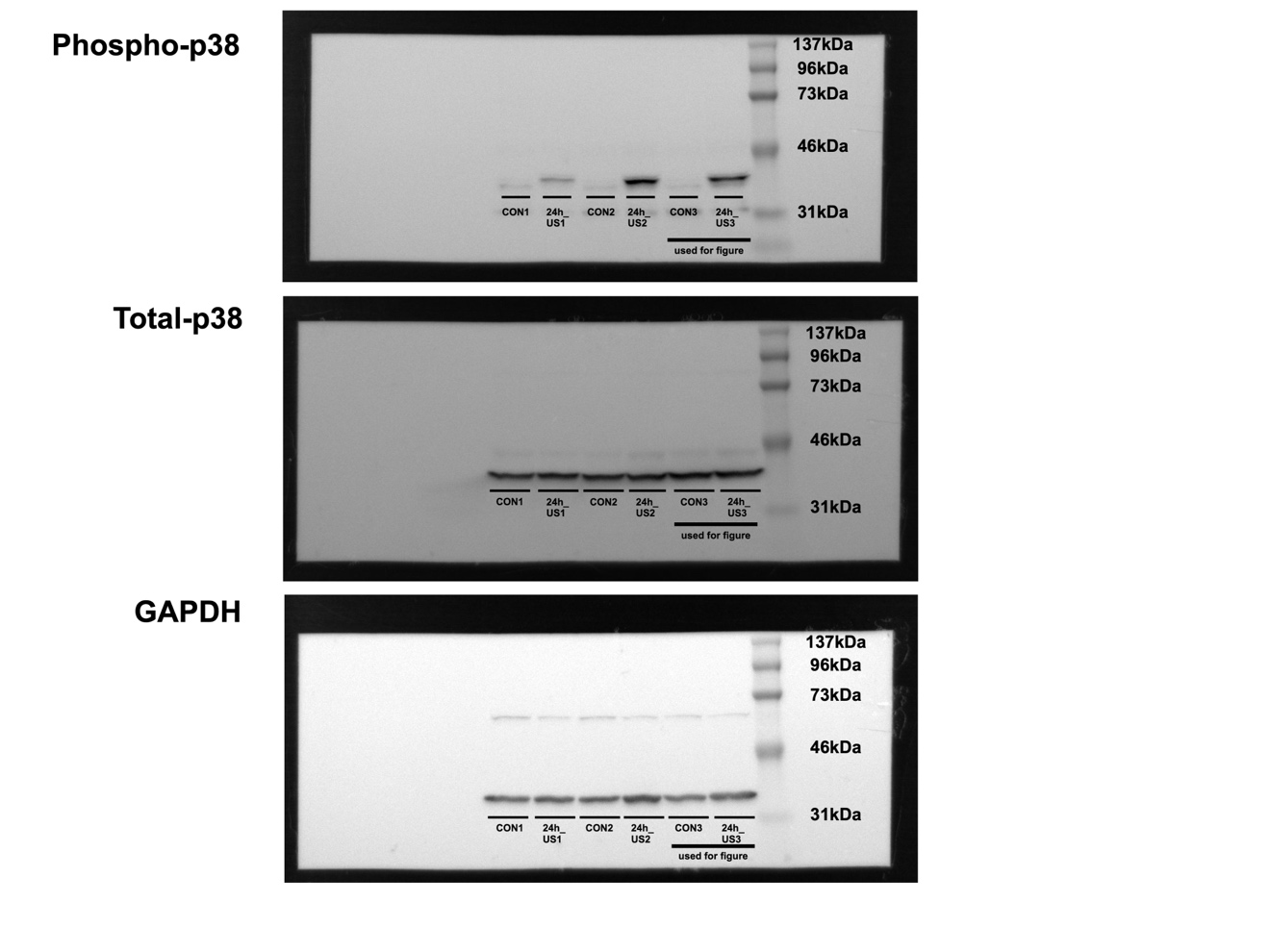
**

The full strip charts for Phospho-p38, Total-p38 and GAPDH
